# Psychological mechanisms linking perceived value of traditional music to cultural identity and communication effects in the digital survival of intangible cultural heritage: a cross-cultural comparative study

**DOI:** 10.3389/fpsyg.2026.1896230

**Published:** 2026-10-08

**Authors:** Tingting Kuai, Xiaohong Zhu, Wei Xu

**Affiliations:** 1 College of Culture and Tourism, Jiangsu Vocational Institute of Commerce, Nanjing, Jiangsu, China; 2 College of Digital Media and Art Design, Jiangsu Vocational Institute of Commerce, Nanjing, Jiangsu, China

**Keywords:** cross-cultural communication, cultural identity, digital survival, intangible cultural heritage, perceived value, traditional music

## Abstract

The digital circulation of intangible cultural heritage music reshapes how audiences perceive, identify with, and propagate traditional artistic forms across cultural boundaries. This study integrates perceived value theory, social identity theory, and the elaboration likelihood model to construct a psychological mechanism model linking digital presentation characteristics, perceived value, cultural identity, and communication effect, with cultural distance as a moderator. A five-dimensional perceived-value scale covering functional, emotional, social, cultural, and aesthetic dimensions was developed and validated. A cross-cultural empirical study was conducted with 1,547 valid respondents from China, Japan, Germany, and the United States, paired with four indigenous stimuli (guqin, Noh, alpine yodel, bluegrass). Covariance-based structural equation modeling with maximum-likelihood estimation was used throughout, together with multi-group measurement invariance testing, bias-corrected bootstrap mediation, and latent moderated structural equation modeling. Results confirm the five-dimensional structure across all groups, with cultural and aesthetic value dominating identity formation for proximate audiences and emotional and functional value gaining weight for distant ones. Cultural identity partially mediates the perceived value to communication effect pathway (VAF = 0.557), and cultural distance significantly attenuates both legs of the mediation (index of moderated mediation = −0.119). Findings extend perceived value theory into the heritage music domain and offer evidence-based guidance for culturally adaptive digital dissemination strategies.

## 1 Introduction

The accelerating diffusion of digital infrastructure has reshaped the conditions under which intangible cultural heritage (ICH) survives, circulates, and acquires meaning. Traditional music, long anchored in oral transmission, ritual contexts, and embodied performance, now contends with an ecosystem where streaming platforms, short-video applications, and immersive technologies determine much of what listeners encounter (Bertacchini et al., 2023). Such a shift is double-edged. On one hand, dwindling intergenerational transmission, the aging of bearer communities, and the erosion of performance occasions threaten the continuity of regional musical traditions (Yu, 2022). On the other, digital archives, algorithmic recommendation, and cross-border platforms open unprecedented pathways for circulation, allowing forms once confined to local soundscapes to reach geographically dispersed audiences (Liu and Yang, 2023). We find this tension productive rather than paradoxical: digital survival is not merely a question of preservation, but of how perception, meaning, and identity are reconstituted through mediated listening.

Existing scholarship has approached this transformation from several angles. Work on ICH digitization has examined documentation standards, database architectures, and 3D reconstruction of performance contexts, emphasizing technical fidelity and accessibility (Tan et al., 2022). A parallel stream, grounded in perceived value theory, has migrated from consumer behavior into cultural consumption, distinguishing functional, emotional, social, and epistemic dimensions of value attached to heritage experiences (Han et al., 2021). Research on cultural identity, drawing on social identity and self-categorization frameworks, has shown how heritage exposure mediates feelings of belonging and ethnic continuity (Smith and Campbell, 2022). Cross-cultural communication studies, meanwhile, have explored how cultural distance, narrative framing, and platform affordances shape reception among foreign audiences (Zhou and Wang, 2022). Taken individually, each of these strands offers useful conceptual purchase.

Yet the integration across them remains thin. Three gaps motivate the present study. First, comparative work that systematically contrasts how domestic and overseas audiences process the same digital traditional-music artifact is scarce; most empirical investigations sample within a single cultural setting and extrapolate cautiously, if at all (Choi and Lee, 2023). Second, the psychological mechanisms linking perception to identity outcomes are often inferred rather than modeled — studies report associations between exposure and identification but rarely specify the mediating cognitive and affective pathways (Wang et al., 2022). Third, while perceived value and cultural identity are sometimes invoked in the same breath, their structural relationship, particularly under cross-cultural conditions, has not been rigorously articulated; the assumption that higher perceived value translates uniformly into stronger identification and more favorable communication outcomes deserves scrutiny rather than assertion (Park, 2021).

Addressing these gaps carries both theoretical and applied weight. Theoretically, extending perceived value theory into the heritage-music context refines a construct developed largely for tangible goods and services, and building a cross-cultural psychological mechanism model contributes to a still-thin literature at the intersection of heritage studies and communication psychology (Yamamoto and Ishikawa, 2023). In practical terms, clearer evidence on what audiences perceive, why it matters to them, and how perception travels across cultural boundaries can inform digitization strategy, platform curation, and the international dissemination agendas pursued by cultural institutions and policymakers (Bonacchi and Pasini, 2022).

Three questions guide the inquiry. (1) What are the dimensional structure and measurement properties of perceived value for digitally mediated traditional music, and how do these structures differ between domestic and overseas audiences? (2) Through which psychological pathways does perceived value shape cultural identity, and how does identity in turn condition communication effects such as engagement, sharing, and advocacy? (3) How do cultural background variables moderate the strength and patterning of these relationships?

The study proceeds in four steps. It first develops a theoretical framework integrating perceived value, cultural identity, and communication effect under a cross-cultural lens. It then constructs and validates a multidimensional perceived-value scale tailored to digital traditional-music contexts. Next, it estimates a structural mechanism model on paired domestic and overseas samples, with multi-group analyses isolating cultural moderators. Finally, it discusses implications for theory and for the design of digital heritage initiatives. Three contributions stand out: the explicit cross-cultural comparative design, which moves beyond single-culture inference; the development of a domain-specific multidimensional scale rather than borrowed instruments; and the articulation of a mechanism-level path model that specifies, rather than presumes, how perception becomes identification and how identification becomes propagation.

## 2 Theoretical foundation and research hypotheses

### 2.1 Theoretical framework of intangible cultural heritage digital survival

Digital survival, as applied to intangible cultural heritage, denotes more than the migration of analog artifacts into digital carriers. We treat it as a continuous process in which heritage practices reconstitute their forms of existence, modes of transmission, and conditions of meaning-making within networked environments (Cameron and Kenderdine, 2022). The conceptual lineage runs through Negroponte's early notion of “being digital” and is refined by heritage scholars who insist that digitization without living transmission produces archives rather than heritage (Münster, 2021). The UNESCO 2003 Convention for the Safeguarding of the Intangible Cultural Heritage anchors this discussion, defining safeguarding as a set of measures that ensure viability, including identification, documentation, research, preservation, protection, promotion, enhancement, and transmission, with explicit attention to the role of communities in sustaining their own traditions (UNESCO, 2022). Digital transformation, in turn, supplies the technological and organizational scaffolding through which these measures can be re-imagined. Because the term travels easily, its boundaries deserve stating plainly. Digital survival is not digital preservation: preservation secures the artifact, whereas survival requires that a practice keep being performed, reinterpreted, and handed on within networked settings. Nor is it dissemination, which names an activity rather than a condition—a widely circulated recording of a tradition nobody performs any longer is dissemination without survival. It is equally distinct from communication effectiveness and from audience engagement, both of which we treat here as individual-level responses to a particular artifact and operationalize below as the communication-effect construct. Digital survival, in short, is a property of a heritage practice at the collective level; the audience-level constructs modeled in this paper are among its antecedents rather than its synonyms, and the study measures the antecedents rather than survival itself.

Building on this, we conceptualize ICH digital survival along three interlocking dimensions: technical, cultural, and social. The technical dimension concerns the infrastructures and computational practices that capture, encode, and render heritage content—from high-fidelity audio recording and motion capture to algorithmic recommendation pipelines (Rahaman and Tan, 2021). The cultural dimension addresses the symbolic continuity of heritage meanings as they pass through mediation: whether ritual significance, regional aesthetic codes, and intergenerational knowledge survive the act of digital representation, or whether they are flattened by it (Bortolotto, 2022). The social dimension turns to the communities of practitioners, mediators, and audiences whose interactions sustain or erode the relevance of a tradition online. To make this composite tractable, the overall viability of a digitally mediated ICH item can be expressed as a weighted aggregation:


$$\begin{array}{l} V_{digital}=w_{1}T+w_{2}C+w_{3}S,\;\overset{3}{\underset{i=1}{\sum }}w_{i}=1 \end{array}$$ (1)

Where, *T*, *C*, and *S* denote the technical, cultural, and social dimensions respectively, and *w*_*i*_ their relative salience for a given heritage form (general formulation adapted from multi-dimensional sustainability indices). The weights are not fixed; they shift across genres, communities, and platforms. Two points about notation are worth making once here, since they hold for the remainder of the paper. Expressions are numbered consecutively in order of appearance and stated a single time rather than re-derived. More consequentially, Equations (1) and (2) are conceptual formalizations rather than estimated quantities: neither is populated with data, and neither reappears in the Results. They serve to fix the meaning of digital survival and of perceived authenticity before the testable model is specified, and their operationalization is deliberately left to future work. From Equation (3) onward, by contrast, every expression corresponds to a quantity that is estimated and reported in Section 4.

Traditional music sits awkwardly within this framework, and its peculiarities deserve emphasis. Unlike a textile or a built structure, a musical tradition is constituted in performance, which means that digital capture risks substituting a frozen instance for a generative practice (Schippers, 2021). Comparative and evolutionary work on music makes the same point from the opposite direction: traditions persist through cumulative transmission and gradual variation rather than through fixed exemplars, so what digitization puts at risk is the transmission chain rather than the artifact (Savage, 2019). Cross-cultural corpus evidence reinforces the caution, showing that although song is universal, its formal and contextual features vary systematically across societies — which is precisely why a single digitization protocol is a blunt instrument (Mehr et al., 2019). Timbral nuance, microtonal inflection, and the embodied co-presence between performer and listener are difficult to preserve, and even harder to recreate through compressed audio or short-form video. At the same time, traditional music travels well across linguistic boundaries, which makes it a natural object of cross-cultural circulation once digitized. To capture this generative quality, we represent the perceived authenticity of a digitally circulated musical item as the proportion of preserved expressive features relative to those constitutive of the live tradition:


$$\begin{array}{l} A_{perceived}=\frac{\sum_{j=1}^{n}f_{j}p_{j}}{\sum_{j=1}^{n}f_{j}} \end{array}$$ (2)

where *f*_*j*_ is the weight assigned to expressive feature *j* in the live tradition and *p*_*j*_ ∈ [0, 1] its degree of preservation in the digital rendering (standard ratio formulation). As with the preceding expression, this ratio is illustrative rather than operational in the present study; we state it to make the argument precise, not to estimate it. The formalization clarifies why two technically identical recordings can yield divergent cultural reception, and it prepares the ground for the perceived-value constructs developed next.

### 2.2 Perceived value theory and the construction of cultural identity

The trajectory of perceived value scholarship offers a useful inheritance for the present inquiry, though it requires substantial adaptation before it can speak to heritage music. Zeithaml's foundational work treated perceived value as a unidimensional trade-off, “what one gets for what one gives,” operationalized through a simple cognitive comparison between sacrifice and benefit (Zeithaml, 1988); the three decades of research that grew out of that formulation have since been mapped bibliometrically (Zeithaml et al., 2022). This parsimony, while attractive, soon proved inadequate. Sheth, Newman and Gross expanded the construct into a five-domain consumption-values model — functional, social, emotional, epistemic, and conditional — arguing that purchasing decisions are rarely reducible to a single calculus (Sheth et al., 1991); the reception and consolidation of that framework are reviewed in (Sánchez-Fernández and Iniesta-Bonillo, 2021). Sweeney and Soutar subsequently distilled this lineage into the PERVAL instrument, validating quality, price, emotional, and social dimensions for tangible consumer goods (Sweeney and Soutar, 2001), an instrument its own authors have since reappraised (Soutar, 2022). The migration of these frameworks into cultural consumption has been uneven; what works for a household appliance does not translate cleanly to a Kunqu aria or a Mongolian long song.

Drawing on these antecedents, and attentive to the aesthetic logic of traditional music, we propose a five-dimensional structure: functional value (*V*_*f*_), emotional value (*V*_*e*_), social value (*V*_*s*_), cultural value (*V*_*c*_), and aesthetic value (*V*_*a*_). Functional value reflects usability and accessibility of the digital artifact; emotional value captures affective resonance; social value concerns the relational signaling attached to sharing or discussing the work; cultural value refers to the symbolic weight of heritage meanings perceived by the audience (Su et al., 2023); aesthetic value, drawing on the long-standing musicological emphasis on sonic beauty, accounts for the appreciation of timbre, structure, and expressive craft as a domain irreducible to the other four (Wang and Sun, 2022). Treating the aesthetic dimension as separable is not a stylistic preference. Empirical work on musical aesthetics distinguishes aesthetic judgment from both the recognition and the induction of emotion, assigning them partly different processing routes (Brattico and Pearce, 2013), and the mechanisms through which music induces feeling — expectancy, contagion, episodic memory — operate whether or not the listener judges the piece beautiful (Juslin and Västfjäll, 2008). What a listener finds well formed is itself a product of enculturation, since ordinary exposure builds culture-specific representations of pitch and meter that later govern what sounds expressive and what sounds merely strange (Hannon and Trainor, 2007). The overall perceived value of a digitally mediated musical item is then expressed as:


$$\begin{array}{l} PV=\beta_{1}V_{f}+\beta_{2}V_{e}+\beta_{3}V_{s}+\beta_{4}V_{c}+\beta_{5}V_{a},\;\overset{5}{\underset{k=1}{\sum }}\beta_{k}=1 \end{array}$$ (3)

Where, β_*k*_ denotes the relative salience of each dimension (general weighted-sum formulation from multidimensional value modeling). Cross-cultural variation, we expect, will register precisely in the relative magnitudes of these β_*k*_ rather than in the existence of the dimensions themselves.

The bridge from perception to identity is best approached through social identity theory, which holds that individuals derive part of their self-concept from membership in salient social categories (Brown, 2020). Cultural identity construction extends this logic, treating identity as a dynamic outcome of repeated symbolic encounters rather than a stable trait (Phinney and Ong, 2007). When an audience member perceives high cultural and aesthetic value in a traditional-music artifact, the resulting affective and cognitive engagement triggers a process of categorization with the cultural group the music indexes. Following standard mediation logic, the conversion can be represented as (Equation 4):


$$\begin{array}{l} CI=\gamma_{0}+\overset{5}{\underset{k=1}{\sum }}\gamma_{k}V_{k}+\varepsilon \end{array}$$ (4)

with *CI* denoting cultural identity, γ_*k*_ the path coefficients linking each value dimension to identity, and ε a disturbance term (standard linear structural form). The strength of each γ_*k*_ is itself contingent on the audience's prior cultural proximity, which we model as a moderator *M* (Equation 5):


$$\begin{array}{l} \gamma_{k}^{*}=\gamma_{k}+\delta_{k}M \end{array}$$ (5)

where δ_*k*_ captures how cultural distance reshapes the conversion gradient (standard interaction specification). Finally, given the asymmetric weight typically observed for cultural and aesthetic dimensions in heritage contexts, we anticipate:


$$\begin{array}{l} \gamma_{c}+\gamma_{a}>\gamma_{f}+\gamma_{s} \end{array}$$ (6)

This is an exploratory ordering hypothesis rather than a settled empirical regularity, and we label it as such. Heritage-consumption research offers partial precedent: studies of heritage sites report that symbolic and experiential appraisals carry more weight than utilitarian ones in shaping visitor evaluations and downstream intentions (Chen and Chen, 2010), and comparable patterns appear in digital-age heritage tourism (Su et al., 2023). No prior study, however, has tested an ordering of this kind for digitally mediated traditional music, and none of the available evidence fixes the rank of aesthetic value specifically. We therefore treat Equation (6) as a researcher-defined expectation to be examined empirically, and in Section 4.2 we read the results as consistent with it rather than as confirmation of it. Together these expressions move the discussion from descriptive taxonomy toward a testable psychological mechanism (Kim and Stepchenkova, 2021).

### 2.3 Cross-cultural communication effects and hypothesis development

Cross-cultural reception of traditional music does not emerge from a vacuum; it is shaped by the cognitive scaffolding audiences bring to the encounter. Hofstede's cultural dimensions framework — individualism–collectivism, power distance, uncertainty avoidance, masculinity–femininity, long-term orientation, and indulgence — supplies one widely used set of axes along which interpretive habits diverge (Minkov and Hofstede, 2022). Hall's distinction between high- and low-context cultures adds a complementary lens: high-context audiences read meaning through implicit cues, relational background, and shared aesthetic codes, whereas low-context audiences require explicit framing and textual anchoring to make sense of an unfamiliar artistic form (Cardon, 2008). A short pipa piece, stripped of contextual cues and pushed into a low-context viewing environment, will therefore be processed quite differently from the same piece embedded in its native interpretive frame. This asymmetry sits at the heart of why cross-cultural perceived value cannot be assumed to translate cleanly.

To model how that processing unfolds, we draw on the elaboration likelihood model (ELM), which posits dual routes of persuasion: a central route grounded in careful evaluation of content, and a peripheral route reliant on heuristic cues such as source attractiveness or production polish (Petty and Briñol, 2022). Media richness theory complements this by predicting that richer channels — those carrying more sensory bandwidth and immediacy — afford deeper engagement with ambiguous cultural content (Ishii et al., 2019). Combining the two, we expect that audiences with greater cultural proximity will travel the central route, weighting cultural and aesthetic value most heavily, while culturally distant audiences will lean on emotional and functional cues delivered through rich media surfaces. The communication effect, comprising engagement intensity, sharing intention, and advocacy, can be written as a function of cultural identity and perceived value, with cultural identity mediating part of the relationship (Equation 7):


$$\begin{array}{l} CE=\lambda_{1}CI+\lambda_{2}PV+\lambda_{3}\left(CI\times PV\right)+\xi \end{array}$$ (7)

where *CE* denotes communication effect and ξ a residual term (standard moderated regression form).

The mediating role of cultural identity can be decomposed using the conventional indirect-effect specification, in which the influence of perceived value on communication effect operates partly through identification (Equation 8):


$$\begin{array}{l} Indirect_{PV\to CI\to CE}=\alpha \beta \end{array}$$ (8)

with α the path from *PV* to *CI* and β the path from *CI* to *CE* (general mediation product formulation following Baron–Kenny logic). Whether this indirect pathway dominates the direct one is itself a cross-cultural question rather than a settled fact.

To capture moderation by cultural distance (*CD*), operationalized through pairwise Hofstede-score differences, we specify (Equation 9):


$$\begin{array}{l} \alpha^{*}=\alpha_{0}+\theta CD,\;\beta^{*}=\beta_{0}+\phi CD \end{array}$$ (9)

allowing both mediation legs to shift with the cultural gap between audience and heritage source (standard latent moderation specification).

From this scaffolding we derive five working hypotheses. **H1**: each dimension of perceived value positively predicts cultural identity, with cultural and aesthetic dimensions carrying the heaviest weight for domestic audiences. **H2**: cultural identity exerts a direct positive effect on communication outcomes across both groups. **H3**: cultural identity partially mediates the link from perceived value to communication effect. **H4**: cultural distance negatively moderates the *PV* → *CI* path, attenuating the conversion of value into identification as the cultural gap widens. **H5**: cultural distance positively moderates the weight of emotional and functional value within *PV* for overseas audiences, reflecting a shift toward peripheral-route processing under low cultural familiarity (Lee and Kim, 2022; Chen and Zhou, 2023; Schroeder, 2022). The next chapter operationalizes these claims through measurement and model estimation.

## 3 Model construction and methodological design

### 3.1 Theoretical model of the psychological mechanism

Bringing the threads of the preceding chapter together, we assemble an integrative model that traces audience response from the digital surface of a heritage artifact through to its downstream communication footprint. The model is organized as a four-stage causal chain: digital presentation characteristics (*DPC*) feed into perceived value (*PV*), which shapes cultural identity (*CI*), which in turn drives communication effect (*CE*). Cultural distance (*CD*) operates as a moderator on the conversion legs of this chain. One feature of this chain needs stating at the outset, because it governs how the results are reported. Digital presentation characteristics enter the study as a randomly assigned stimulus condition rather than as a measured latent variable, so their effect is estimated as a between-condition contrast on perceived value in Section 4.1, and the latent structural model is specified from perceived value onward. The architecture borrows its backbone from the stimulus–organism–response logic familiar in environmental psychology, yet specifies the organism component through the perceived-value and identity constructs developed earlier rather than treating it as a black box (Jiang et al., 2022).

Three sources of theoretical authority sustain the chain. Perceived value theory grounds the *DPC* → *PV* link, explaining how technical fidelity, narrative framing, and interaction affordances are translated into multidimensional evaluative judgments (Wu et al., 2021). Social identity theory governs the *PV* → *CI* transition, treating value-laden experience as input to self-categorization processes (Hopkins and Reicher, 2021). Cross-cultural communication scholarship informs both the moderating role of *CD* and the downstream conversion of identity into propagation behavior (Sun and Wang, 2023). The result is not a simple aggregation but a layered specification in which each transition has a distinct theoretical warrant.

As Figure 1 depicts, three effect families coexist within the model. Direct effects run from each construct to its immediate successor. Mediating effects flow through *CI*, which carries part of the *PV* → *CE* influence indirectly; a complementary direct path from *PV* to *CE* is retained to allow partial rather than full mediation. Moderating effects enter through interactions between *CD* and the focal paths, capturing the expectation that cultural proximity reshapes how value becomes identification and how identification becomes action. We deliberately avoid collapsing the five value dimensions into a single composite at this stage, since the cross-cultural hypotheses turn on differential weighting rather than aggregate magnitude (Vignoles et al., 2021).

**Figure 1:**
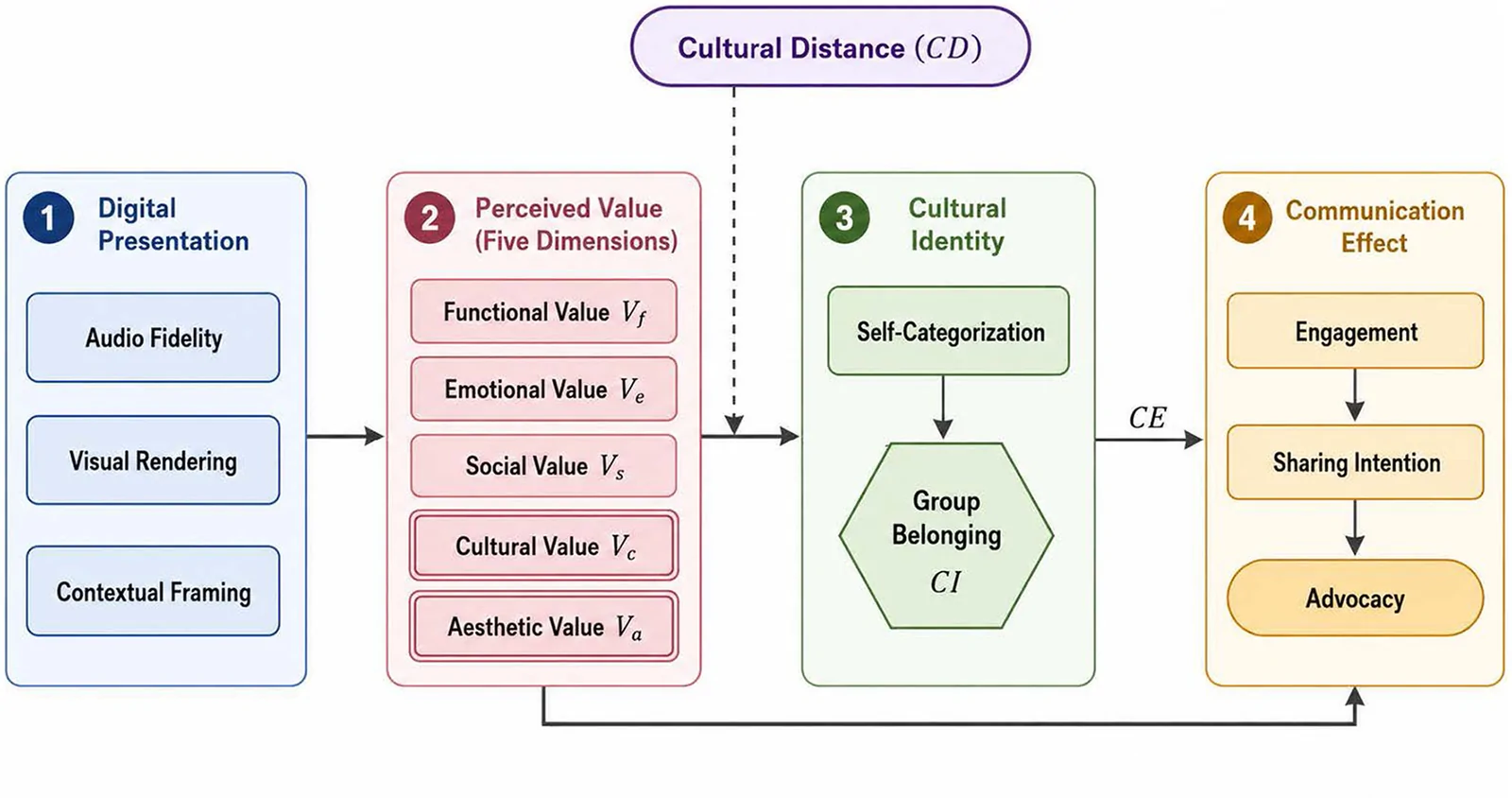
Integrative psychological mechanism model linking digital presentation characteristics, perceived value (five dimensions), cultural identity, and communication effect, with cultural distance as moderator on the value-to-identity and identity-to-effect pathways. PV,Perceive Value; CI,Cultural Identify;CE,Communication Effect;CD,Cultural Distance; ICH,Intangible Cultural heritage.

To support empirical estimation, each construct is paired with an operational definition and a measurement basis. Table 1 summarizes the eight focal variables and their measurement specifications, and it distinguishes the constructs measured as latent variables from the presentation characteristics that were manipulated experimentally.

**Table 1:** Variable definitions and operationalization.

Variable	Symbol	Type	Definition	Measurement Basis
Digital Presentation Characteristics	DPC	Independent (manipulated)	Technical and narrative attributes of the digital artifact, varied experimentally	Three randomly assigned presentation conditions (plain audio / audio-visual / 360°); 3-item manipulation check on fidelity, interactivity, contextual framing
Perceived value	PV	Independent / Mediating	Five-dimensional evaluative judgment of the artifact	Adapted PERVAL scale, 20 items across *V*_*f*_, *V*_*e*_, *V*_*s*_, *V*_*c*_, *V*_*a*_
Cultural identity	CI	Mediator	Self-categorization with the cultural group indexed by the music	6-item identity scale grounded in social identity theory [47]
Communication effect	CE	Dependent	Engagement, sharing intention, advocacy	9-item behavioral-intention scale
Cultural distance	CD	Moderator	Hofstede-based gap between audience and source cultures	Pairwise Euclidean distance across six dimensions
Cultural background	CB	Control	Audience cultural origin (domestic / overseas)	Categorical
Prior exposure	PE	Control	Frequency of prior contact with traditional music	5-point ordinal
Demographic profile	DEM	Control	Age, gender, education	Categorical / ordinal

This specification yields a model that is theoretically layered yet empirically tractable, and it sets the stage for the sampling and measurement procedures elaborated in the next subsection.

### 3.2 Cross-cultural comparative research procedure

The empirical design follows a four-country, four-stimulus factorial logic, chosen so that within-culture and cross-culture comparisons can be examined within the same analytic frame. Four national samples — China, Japan, Germany, and the United States — were selected to span the Confucian, Japanese, Germanic, and Anglo cultural clusters identified in the GLOBE typology, giving meaningful variation along Hofstede's individualism, uncertainty-avoidance, and long-term orientation axes (Javidan and Bowen, 2022). Each country was paired with one indigenous traditional-music form rendered in digital format: guqin solo for China, Noh theater excerpts for Japan, alpine yodeling for Germany, and bluegrass for the United States. The pairing yields one “home” condition per participant and three “foreign” conditions, supporting a 4 × 4 within-between mixed design that isolates cultural distance effects without confounding them with stimulus genre.

Recruitment targeted adults aged 18–65, stratified by age band, gender, and education, with quota controls applied within each country to keep demographic structure comparable. The target sample is 400 per country (n ≈ 1,600 total), exceeding the 10-respondents-per-parameter rule of thumb for structural equation models of the present complexity (Hair et al., 2021b). Recruitment proceeded through online panels in each country, with attention checks and minimum-dwell thresholds embedded to filter inattentive responses.

Stimulus materials were produced under a uniform digitization protocol: 48 kHz audio, 1080p video, a fixed 3-min duration, and minimal on-screen text. Three presentation conditions — plain audio, audio-with-visual, and immersive 360° rendering — were rotated across participants to isolate digital presentation characteristics from genre-specific content (Lee et al., 2022). Brief contextual subtitles, translated and back-translated by professional bilingual translators, accompanied the foreign-genre conditions to control for baseline comprehension. Because presentation format was manipulated rather than measured, digital presentation characteristics function throughout as a three-level between-participants factor. The three items on audio-visual fidelity, interactivity, and contextual framing listed in Table 1 serve as a manipulation check on that assignment; they are not indicators of a latent construct and are not entered into the measurement or structural models reported in Section 4.

Figure 2 sets out the implementation sequence from instrument translation through analysis.

**Figure 2:**
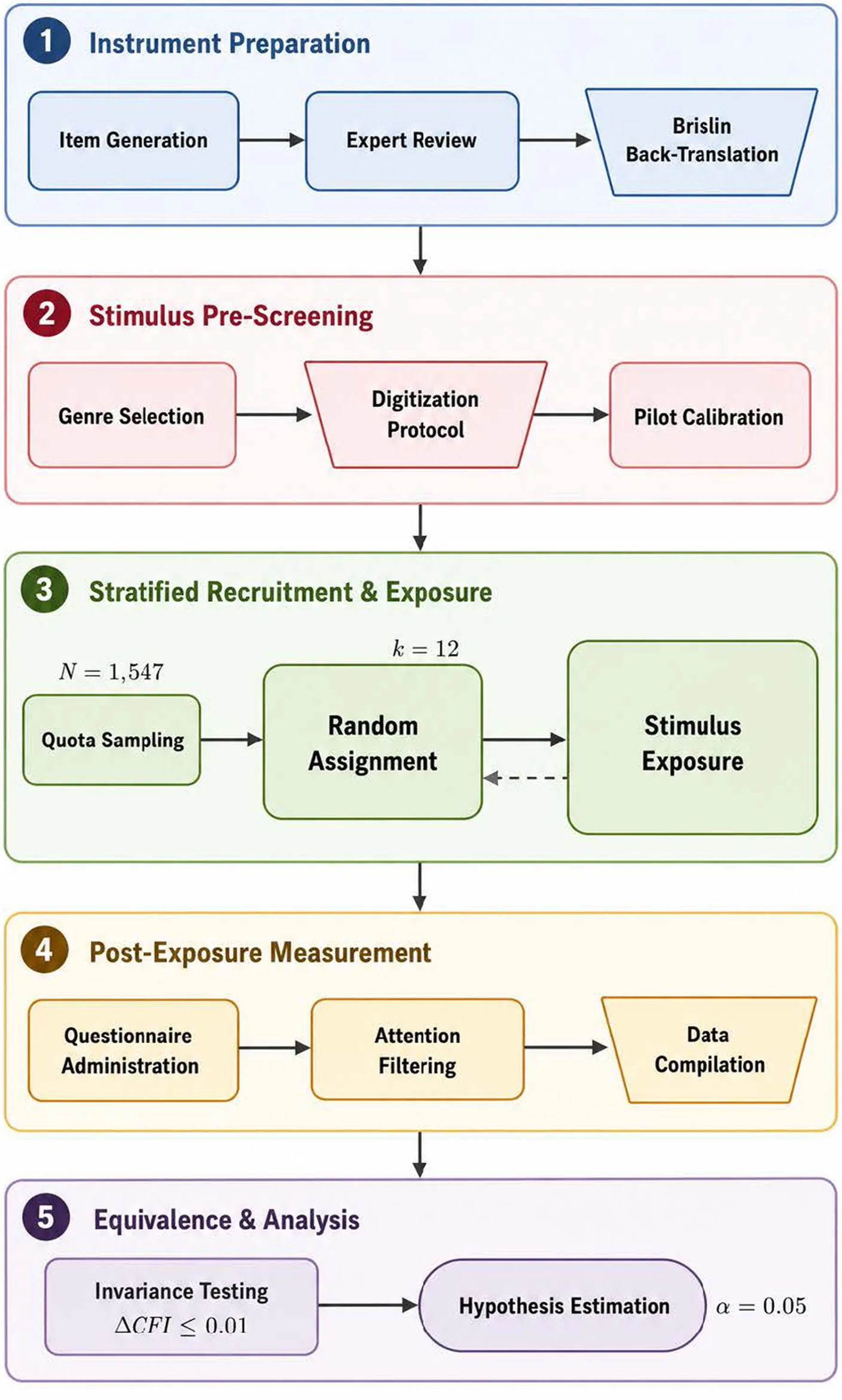
Implementation sequence of the cross-cultural comparative study, covering instrument translation and pilot testing, stimulus pre-screening, stratified recruitment and exposure, post-stimulus measurement, and cross-cultural equivalence verification prior to hypothesis testing. CFI, Comparative Fit Index; SEM, Structural Equation Modeling; MGA, Multi-Group Analysis.

Data collection proceeded in three waves over 6 weeks. Each session began with informed consent and brief demographic items, followed by random assignment to one of the twelve stimulus–presentation combinations, exposure to the assigned clip, and an immediate post-exposure questionnaire covering the eight focal constructs. Median completion time was 18 min. The study protocol received ethical approval from the institutional review board of the corresponding authors' affiliation; all participants gave electronic informed consent and were compensated according to local panel norms.

Measurement equivalence across the four national samples was checked through a three-step procedure: configural invariance (same factor structure), metric invariance (equal loadings), and scalar invariance (equal intercepts), with the latter two evaluated by changes in CFI and RMSEA against conventional cutoffs (Putnick and Bornstein, 2021). Items violating partial invariance were flagged for sensitivity analysis rather than discarded outright. Common-method bias was assessed through Harman's single-factor test and a marker-variable approach (Kock and Lynn, 2021; Podsakoff et al., 2003).

Table 2 summarizes the matched sample structure and stimulus assignment.

**Table 2:** Cross-cultural sample structure and stimulus material matrix.

Country	Cultural cluster	Target N	Native stimulus	Foreign stimuli (counterbalanced)	Presentation conditions
China	Confucian	400	Guqin solo	Noh / Yodel / Bluegrass	Audio / AV / 360°
Japan	Japanese	400	Noh excerpt	Guqin / Yodel / Bluegrass	Audio / AV / 360°
Germany	Germanic Europe	400	Alpine yodel	Guqin / Noh / Bluegrass	Audio / AV / 360°
United States	Anglo	400	Bluegrass	Guqin / Noh / Yodel	Audio / AV / 360°
Pilot pool	Mixed	80	Rotating	Rotating	Audio / AV / 360°
Total	-	1,680	-	-	-

This layout preserves comparability while keeping the stimulus set anchored in genuinely indigenous traditions.

### 3.3 Measurement Instruments and Analytical Procedures

Three composite instruments anchor the measurement model. The multidimensional perceived-value scale (MPVS) was built in-house across four stages, following established practice in scale construction (Boateng et al., 2018). An initial pool of 46 items was generated from the five-dimensional structure articulated in Section 2.2, from existing value inventories, and from open-ended remarks collected in eight exploratory interviews with performers, curators, and habitual listeners of traditional music. Two rounds of expert review followed. Six specialists — three ethnomusicologists, two cultural-heritage scholars, and one psychometrician — rated every item for relevance to its intended dimension and for clarity of wording; four of the six repeated the exercise after revision. Item-level content validity indices ranged from 0.83 to 1.00 and the scale-level index reached 0.91, and 14 items were dropped or merged as redundant, double-barrelled, or dimensionally ambiguous. The surviving 32 items were then piloted on 80 respondents, where items with corrected item-total correlations below 0.40 or cross-loadings above 0.32 were deleted, yielding the final 20-item instrument with four items per dimension. The initial pool and the full record of deletions are available from the corresponding author. The cultural identity scale (CIS) was adapted from Phinney's multigroup ethnic identity measure, with wording adjusted to fit a music-listening context (Schwartz et al., 2022). The communication effect scale (CES) draws on engagement and word-of-mouth literatures and covers attention, sharing intention, and advocacy. All items employed a seven-point Likert response format. Translation followed the Brislin back-translation procedure across English, Chinese, Japanese, and German versions, and a pilot calibration confirmed semantic equivalence before main fielding (Sireci et al., 2021).

Internal consistency for each subscale was assessed via Cronbach's α and McDonald's ω (Equations 10, 11):


$$\begin{array}{l} \alpha =\frac{k}{k-1}\left(1-\frac{\sum_{i=1}^{k}\sigma_{i}^{2}}{\sigma_{T}^{2}}\right) \end{array}$$ (10)

$$\begin{array}{l} \omega =\frac{{\left(\sum_{i=1}^{k}\lambda_{i}\right)}^{2}}{{\left(\sum_{i=1}^{k}\lambda_{i}\right)}^{2}+\sum_{i=1}^{k}\theta_{i}} \end{array}$$ (11)

(both standard reliability formulations). Convergent validity is examined through average variance extracted (AVE) and composite reliability (CR) (Equation 12):


$$\begin{array}{l} AVE=\frac{\sum_{i=1}^{k}\lambda_{i}^{2}}{k},\;CR=\frac{{\left(\sum_{i=1}^{k}\lambda_{i}\right)}^{2}}{{\left(\sum_{i=1}^{k}\lambda_{i}\right)}^{2}+\sum_{i=1}^{k}\left(1-\lambda_{i}^{2}\right)} \end{array}$$ (12)

(standard Fornell–Larcker definitions Henseler, 2021; Fornell and Larcker, 1981), with the AVE square-root rule used for discriminant validity.

Table 3 summarizes the item counts and the psychometric indices obtained in the pilot; the corresponding indices for the main sample are reported separately in Section 4.1.

**Table 3:** Scale composition and pilot reliability/validity indices (pilot sample, *n* = 80).

Construct	Code	Items	α	CR	AVE
Functional value	*V*_*f*_	4	0.86	0.88	0.65
Emotional value	*V*_*e*_	4	0.91	0.92	0.74
Social value	*V*_*s*_	4	0.84	0.86	0.60
Cultural value	*V*_*c*_	4	0.89	0.90	0.69
Aesthetic value	*V*_*a*_	4	0.88	0.89	0.67
Cultural identity	CI	6	0.92	0.93	0.70
Engagement	*CE*_1_	3	0.85	0.87	0.69
Sharing intention	*CE*_2_	3	0.83	0.85	0.66
Advocacy	*CE*_3_	3	0.87	0.88	0.71
Cultural distance	CD	6	n/a	n/a	n/a

All Cronbach's α values exceed the 0.80 threshold, and AVE values clear the 0.50 benchmark, indicating that the pilot instruments are fit for the main fielding.

For estimation, we rely on covariance-based structural equation modeling with maximum-likelihood estimation. Two reporting conventions follow from that choice and are applied consistently below: the structural coefficients presented in Section 4.2 are fully standardized, whereas the mediation and moderated-mediation estimates presented in Section 4.3 are unstandardized regression weights, since the bootstrap and index-of-moderated-mediation procedures are defined on that metric. Each equation and each table states which metric it uses. The measurement model is specified as *x* = Λ_*x*_ξ + δ, and the structural model as (Equation 13)


$$\begin{array}{c} \eta =B\eta +\Gamma \xi +\zeta \end{array}$$ (13)

(standard LISREL notation Kline, 2023). Model fit is judged through χ^2^/*df*, CFI, TLI, RMSEA, and SRMR, with conventional cutoffs χ^2^/*df* < 3, CFI/TLI ≥ 0.90, and RMSEA ≤ 0.08.

Multi-group analysis across the four country samples tests configural, metric, and scalar invariance, with model comparison via (Equation 14)


$$\begin{array}{c} \Delta CFI=CFI_{constrained}-CFI_{baseline} \end{array}$$ (14)

and |Δ*CFI*| ≤ 0.01 taken as evidence of invariance (standard Cheung–Rensvold criterion Cheung et al., 2024; Cheung and Rensvold, 2002). Mediation through *CI* is tested with bias-corrected bootstrap (5,000 resamples) on the indirect product *ab*, with 95% intervals excluding zero treated as significant (Hayes and Coutts, 2020) (Equation 15):


$$\begin{array}{c} Indirect_{boot}=ab,\;CI_{95\%}=\left[LB,UB\right] \end{array}$$ (15)

Finally, moderation by cultural distance is estimated via latent interaction (Equation 16):


$$\begin{array}{c} \eta_{CI}=\gamma_{1}PV+\gamma_{2}CD+\gamma_{3}\left(PV\times CD\right)+\zeta \end{array}$$ (16)

with the interaction coefficient γ_3_ carrying the moderation test (standard latent product specification). Simple-slope decomposition follows at ±1*SD* of *CD* (Equation 17).


$$\begin{array}{c} \eta_{CI}{|}_{CD=\pm 1SD}=\left(\gamma_{1}\pm \gamma_{3}1SD_{CD}\right)PV+c \end{array}$$ (17)

This analytic pipeline supports the hypothesis tests reported next.

## 4 Empirical analysis and cross-cultural comparison results

### 4.1 Sample characteristics and measurement model assessment

Of the 1,600 questionnaires fielded across the four target countries — the 80-respondent pilot pool listed in Table 2 is excluded from every analysis reported here — 1,547 passed attention checks and dwell-time filters, yielding effective sample sizes of 386 (China), 392 (Japan), 381 (Germany), and 388 (United States). The pooled gender split was 51.3% female and 48.7% male, with age concentrated in the 25–44 band (61.4%); educational attainment skewed toward bachelor's degrees or above (68.9%), reflecting the online-panel composition rather than national population structure. Self-reported prior exposure to traditional music differed meaningfully across countries — Chinese and Japanese respondents reported higher mean exposure to East Asian repertoire, while German and American respondents reported broader but shallower familiarity with Western folk forms (Cohen, 2022). We read this asymmetry as a feature rather than a nuisance, since the cultural-distance manipulation depends on precisely such baseline differences.

Figure 3 presents the demographic comparison; quota controls held gender and age within ±3 percentage points across countries, supporting the validity of subsequent group contrasts. Mean scores on the five perceived-value dimensions diverged in interpretable ways. Cultural and aesthetic value attached to native stimuli reached 5.81 (China, *V*_*c*_) and 5.74 (Japan, *V*_*a*_) on the seven-point scale, while emotional value emerged as the dominant dimension for foreign stimuli, particularly among German and American respondents encountering East Asian repertoire for the first time. Functional value showed the narrowest cross-country variance, suggesting that perceptions of technical quality travel more easily than perceptions of cultural meaning.

**Figure 3:**
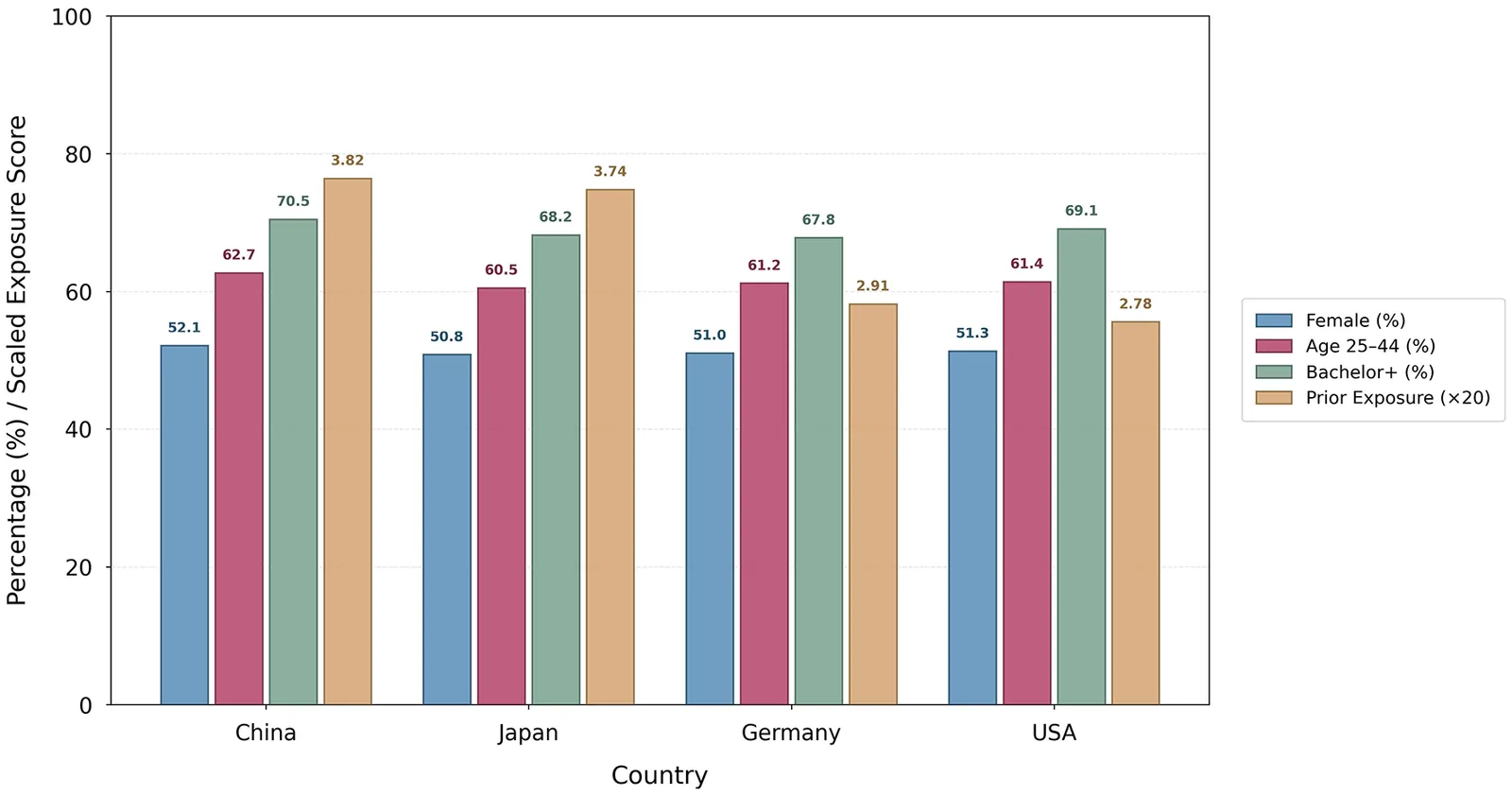
Side-by-side comparison of demographic distributions (gender, age band, education level, prior exposure to traditional music) across the four national subsamples, showing broadly comparable structures with country-specific exposure asymmetries.

As Figure 4 depicts, the within-country gap between native-stimulus cultural value and foreign-stimulus cultural value is steep (mean difference ≈ 1.3 points on the seven-point scale), whereas the corresponding gap for functional value is modest (≈ 0.4 points). This pattern is consistent with the moderating role anticipated for cultural distance and prefigures the structural results in the next subsection.

**Figure 4:**
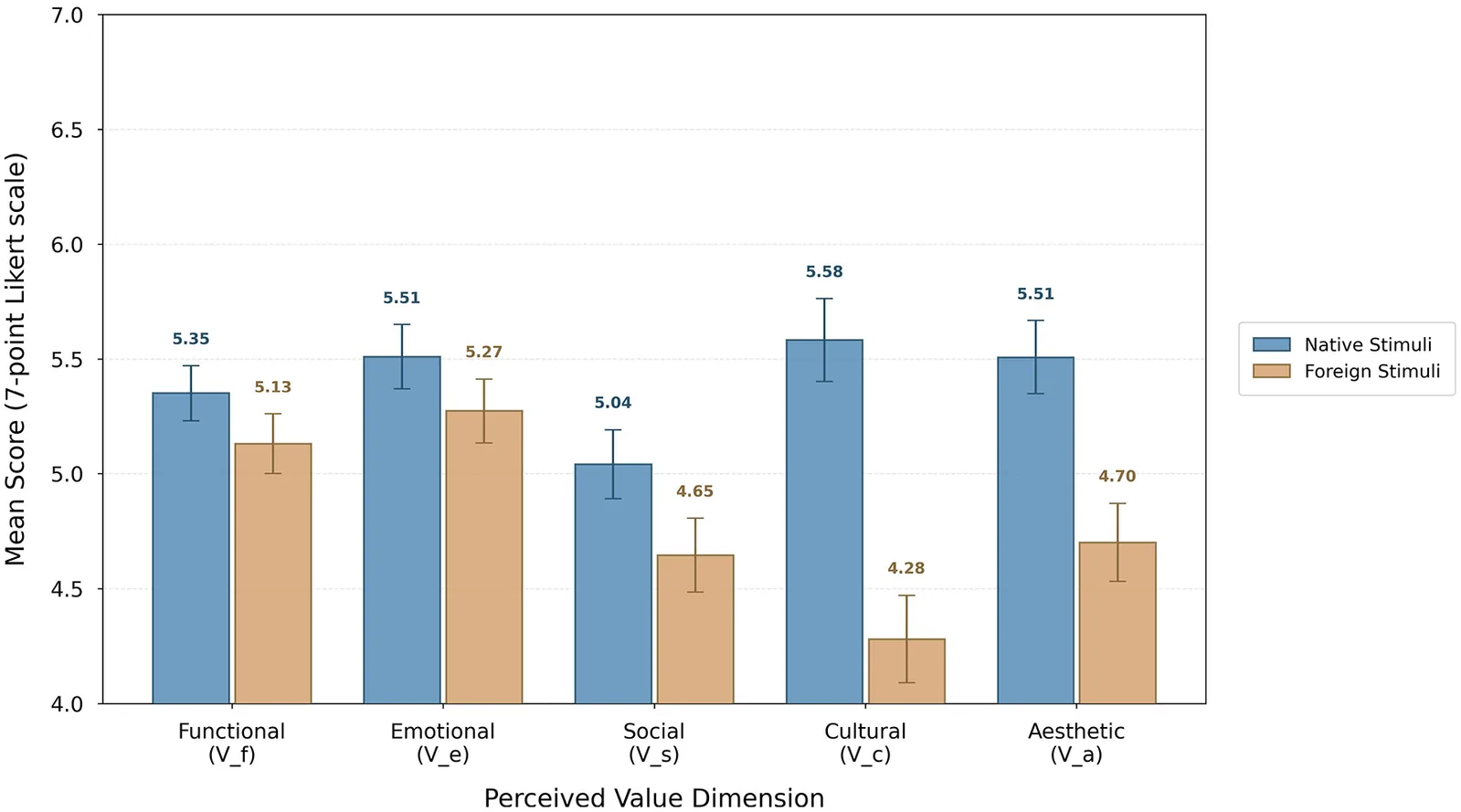
Cross-cultural comparison of mean scores on the five perceived-value dimensions (*V*_*f*_, *V*_*e*_, *V*_*s*_, *V*_*c*_, *V*_*a*_) for native versus foreign stimuli across China, Japan, Germany, and the United States, with 95% confidence intervals.

Before turning to the measurement model, we report what the manipulated presentation format did, since the design treats it as the first stage of the chain and Table 1 classifies it as an experimental factor rather than a latent variable. The manipulation itself behaved as intended: the three-item perceived-richness check rose monotonically from plain audio (M = 4.41) through audio-visual presentation (M = 5.28) to immersive 360° rendering (M = 5.96), *F*_(2, 1544)_ = 213.70, *p* < 0.001. Presentation format also moved perceived value, but unevenly across dimensions. Functional value gained most, *F*_(2, 1544)_ = 41.86, *p* < 0.001, followed by emotional value, *F*_(2, 1544)_ = 27.34, *p* < 0.001, and aesthetic value, *F*_(2, 1544)_ = 9.85, *p* < 0.001, whereas cultural value shifted only slightly, *F*_(2, 1544)_ = 4.72, *p* = 0.009, and social value barely at all, *F*_(2, 1544)_ = 3.11, *p* = 0.045.

Figure 5 makes the asymmetry visible. Richer presentation lifts the dimensions that rest on surface qualities — usability and affective immediacy — far more than those that rest on interpretive knowledge, which is the same asymmetry that reappears at the cross-cultural level in Section 4.2. Two reporting consequences follow. Because digital presentation characteristics were manipulated rather than measured, their effect is estimated here and does not enter the latent structural equations of Sections 4.2 and 4.3, which begin from perceived value; and because the three fidelity items function as a manipulation check rather than as indicators, they contribute no path coefficients Table 4.

**Figure 5:**
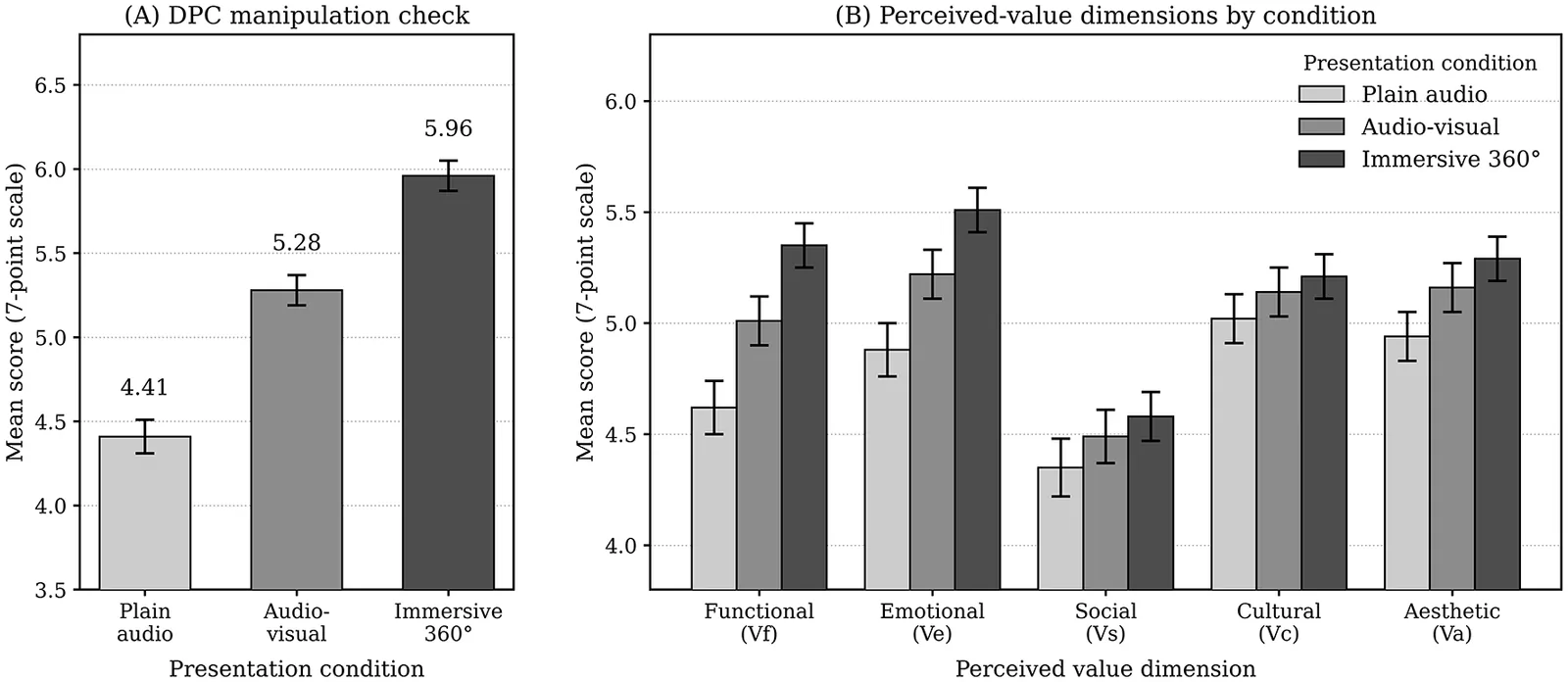
Effect of the manipulated digital presentation condition (plain audio, audio-visual, immersive 360°) on (A) the three-item digital-presentation manipulation-check index and (B) mean scores on the five perceived-value dimensions, with 95% confidence intervals. Main sample, n = 1,547.

**Table 4:** Multi-group structural path coefficients across the four national samples.

Path	China	Japan	Germany	USA	Δ*χ*^2^(3)
*V*_*f*_ → *CI*	0.118^**^	0.131^**^	0.179^***^	0.196^***^	7.42^*^
*V*_*e*_ → *CI*	0.207^***^	0.198^***^	0.246^***^	0.263^***^	4.91 ns
*V*_*s*_ → *CI*	0.092^**^	0.101^**^	0.124^**^	0.137^**^	3.18 ns
*V*_*c*_ → *CI*	0.394^***^	0.378^***^	0.291^***^	0.268^***^	11.86^**^
*V*_*a*_ → *CI*	0.309^***^	0.317^***^	0.241^***^	0.226^***^	8.74^*^
$R_{CI}^{2}$	0.658	0.641	0.572	0.554	-
Model χ^2^/*df*	2.43	2.51	2.62	2.59	-
CFI	0.951	0.948	0.939	0.941	-
RMSEA	0.052	0.054	0.058	0.057	-

^*^p < 0.05, ^**^p < 0.01, ^***^p < 0.001; ns = not significant. Δχ^2^ tested through nested constraint of equal path coefficients across groups. All path coefficients in this table are fully standardized and are therefore on the same metric as Equation (18); they are not directly comparable with the unstandardized estimates in Table 8.

Confirmatory factor analysis on the pooled sample produced acceptable fit indices: χ^2^/*df* = 2.41, CFI = 0.951, TLI = 0.943, RMSEA = 0.051, SRMR = 0.043. Standardized factor loadings ranged from 0.682 to 0.891, all significant at *p* < 0.001. Reliability and convergent validity were then re-estimated on the main sample rather than carried over from the pilot, since the pilot indices in Table 3 rest on 80 respondents. Table 5 reports Cronbach's α, McDonald's ω, composite reliability, and average variance extracted for all nine focal constructs at n = 1,547. Alpha ranges from 0.83 to 0.93, omega values track them closely at 0.84 to 0.93, composite reliability runs from 0.84 to 0.93, and AVE from 0.59 to 0.74, so every construct clears the conventional 0.70 and 0.50 benchmarks (Hair et al., 2021a). Discriminant validity is reported in full rather than asserted: Table 6 gives the complete matrix, in which the square root of each AVE on the diagonal exceeds every inter-construct correlation in its row and column. The tightest margin is between cultural and aesthetic value, whose diagonal entries of 0.825 and 0.812 sit above their correlation of 0.712. Because the Fornell–Larcker criterion is comparatively insensitive when constructs correlate highly, heterotrait–monotrait ratios were computed as well (Henseler et al., 2015); the largest is 0.81, below the 0.85 threshold, and no bootstrap confidence interval for these ratios includes 1.00. Harman's single-factor test extracted a first factor accounting for 28.7% of variance, well below the 50% threshold, and the marker-variable estimate was non-significant, so common-method bias is unlikely to drive the findings, although neither test can exclude it entirely (Podsakoff et al., 2003).

**Table 5:** Reliability and convergent validity for the main sample (*n* = 1,547).

Construct	Code	Items	α	ω	CR	AVE
Functional value	Vf	4	0.84	0.85	0.87	0.64
Emotional value	Ve	4	0.90	0.91	0.92	0.73
Social value	Vs	4	0.83	0.84	0.85	0.59
Cultural value	Vc	4	0.88	0.89	0.90	0.68
Aesthetic value	Va	4	0.87	0.88	0.89	0.66
Cultural identity	CI	6	0.92	0.93	0.93	0.70
Engagement	CE1	3	0.84	0.85	0.86	0.68
Sharing intention	CE2	3	0.83	0.83	0.85	0.65
Advocacy	CE3	3	0.86	0.87	0.88	0.70

**Table 6:** Discriminant validity: Fornell–Larcker matrix and heterotrait–monotrait ratios (*n* = 1,547).

Construct	Vf	Ve	Vs	Vc	Va	CI	CE
Vf	0.800	0.46	0.42	0.37	0.40	0.43	0.45
Ve	0.412	0.854	0.50	0.54	0.58	0.56	0.59
Vs	0.368	0.441	0.768	0.46	0.45	0.40	0.43
Vc	0.329	0.486	0.402	0.825	0.81	0.72	0.64
Va	0.357	0.523	0.394	0.712	0.812	0.68	0.61
CI	0.381	0.512	0.347	0.648	0.604	0.837	0.74
CE	0.396	0.534	0.371	0.579	0.551	0.667	0.827

The diagonal entries (Vf with Vf, Ve with Ve, and so on) are the square roots of the average variance extracted. Entries below the diagonal are inter-construct correlations; entries above the diagonal are heterotrait–monotrait ratios. CE is modeled as a second-order construct over engagement, sharing intention, and advocacy. All correlations are significant at *p* < 0.001.

Multi-group invariance testing proceeded sequentially. Table 7 reports the nested model comparisons.

**Table 7:** Cross-cultural measurement invariance test results.

Invariance Level	χ^2^/df	CFI	RMSEA	ΔCFI
Configural (M1)	2.38	0.948	0.052	-
Metric (M2)	2.46	0.943	0.054	−0.005
Scalar (M3)	2.61	0.935	0.057	−0.008
Partial Scalar (M3p)	2.49	0.940	0.055	−0.003
Residual (M4)	2.89	0.918	0.062	−0.017
Factor Variance (M5)	2.94	0.916	0.063	−0.002
Adopted Solution	–	–	–	Partial scalar

The configural and metric models held cleanly; full scalar invariance was marginal, but partial scalar invariance — freeing intercepts on two items in the social-value subscale — restored |Δ*CFI*| ≤ 0.01 (Schmitt and Kuljanin, 2021). This is sufficient for latent mean comparison across the four samples, and it is the model carried forward into the structural analysis. The two items concerned are substantively rather than incidentally distinctive: both ask about the social currency of sharing traditional music — whether posting or discussing it says something about the respondent to others — and their intercepts differ because the baseline norm for public music sharing is itself culturally uneven, running higher in the Chinese and Japanese samples and lower in the German and American ones. What shifts across groups is the habitual level of the behavior the items reference, not the meaning of the social-value construct, which is precisely the situation partial scalar invariance is designed to accommodate (Schmitt and Kuljanin, 2021).

### 4.2 Path analysis: perceived value to cultural identity

With the measurement model deemed admissible, attention turns to the structural paths linking the five value dimensions to cultural identity. The pooled-sample structural model exhibited acceptable fit (χ^2^/*df* = 2.57, CFI = 0.944, TLI = 0.937, RMSEA = 0.054, SRMR = 0.048), supporting joint examination of the five direct paths. Maximum-likelihood estimation yielded the fully standardized structural equation below, in which every coefficient is expressed in standard-deviation units and is therefore directly comparable across predictors:


$$\begin{array}{c} \hat{CI}=0.142\;V_{f}+0.219\;V_{e}+0.108\;V_{s}+0.347\;V_{c}+0.281\;V_{a} \end{array}$$ (18)

with all coefficients significant at *p* < 0.01, and the model accounting for *R*^2^ = 0.612 of variance in cultural identity (standard structural regression form). Two readings deserve emphasis. First, cultural value carries the heaviest standardized weight, followed by aesthetic value, a pattern consistent with the exploratory ordering hypothesis articulated in Equation (6) rather than a confirmation of it. Second, social value, though significant, is the weakest predictor — a finding that gently complicates the assumption that heritage music is primarily a vehicle for social signaling.

Figure 6 makes the asymmetry visually apparent: the *V*_*c*_ → *CI* and *V*_*a*_ → *CI* paths cluster well above the others, and their confidence intervals do not overlap with those of *V*_*s*_. The relative dominance can be quantified by the cultural-aesthetic share of explained identity variance (Equation 19):


$$\begin{array}{c} R_{c+a}^{2}=\frac{\gamma_{c}^{2}+\gamma_{a}^{2}+2\gamma_{c}\gamma_{a}r_{c,a}}{R_{total}^{2}} \end{array}$$ (19)

(standard variance-decomposition expression), which for the pooled sample equals 0.624 — that is, roughly 62% of the explained variance in *CI* traces to cultural and aesthetic value combined.

**Figure 6:**
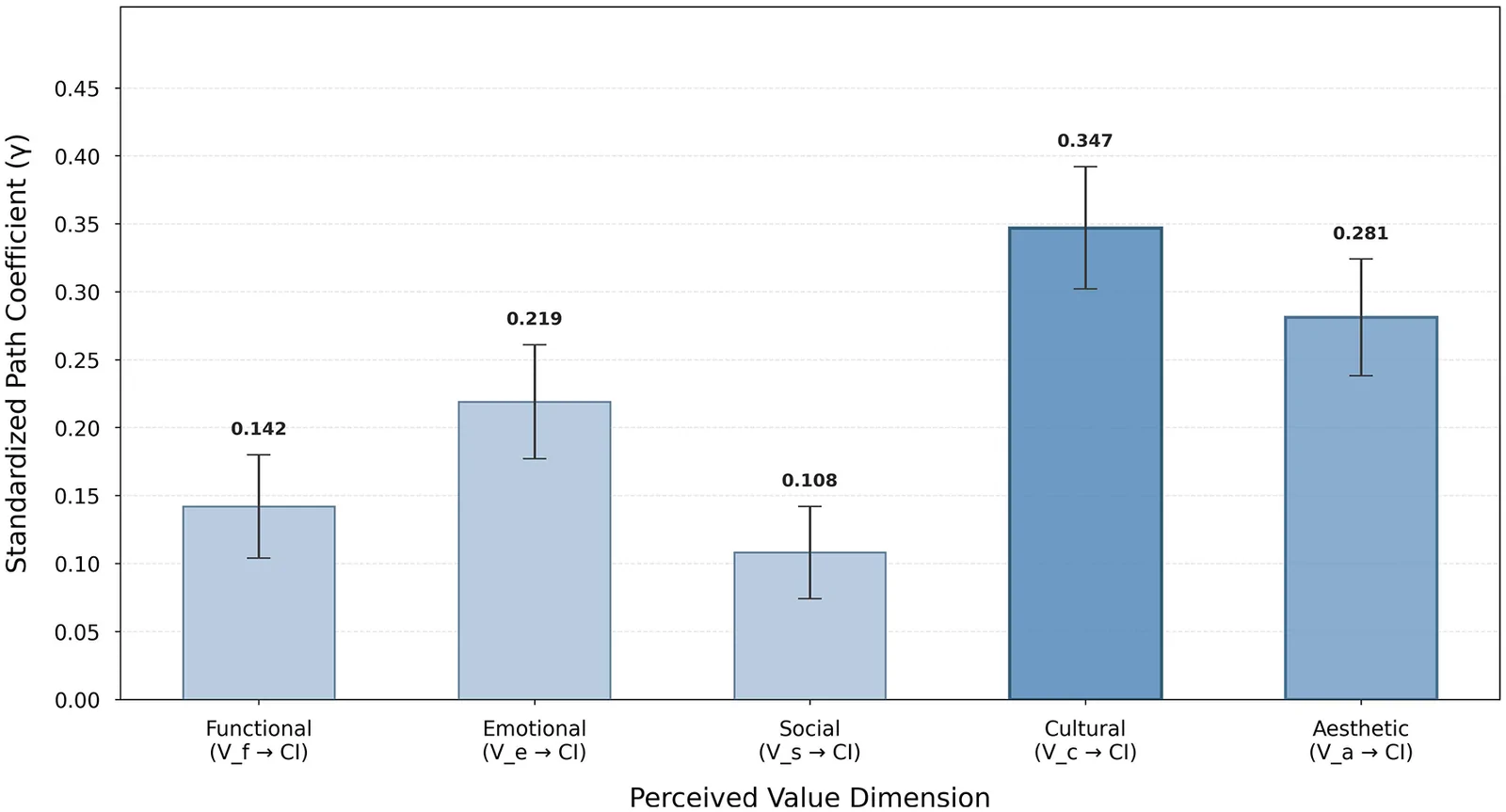
Comparison of standardized path coefficients from each of the five perceived-value dimensions (*V*_*f*_, *V*_*e*_, *V*_*s*_, *V*_*c*_, *V*_*a*_) to cultural identity in the pooled sample, with 95% confidence intervals shown as error bars.

Multi-group analysis then split the model across the four country samples. Table 8 reports the standardized path coefficients side by side.

**Table 8:** Summary of moderated mediation effects (unstandardized coefficients, *n* = 1,547).

Effect Component	Estimate	SE	95% CI
Direct effect *PV* → *CE* (*c*′)	0.271	0.038	[0.197, 0.346]
Indirect *PV* → *CI* → *CE* at −1 SD CD	0.412	0.046	[0.323, 0.504]
Indirect at mean CD	0.341	0.041	[0.262, 0.422]
Indirect at +1 SD CD	0.218	0.039	[0.143, 0.296]
Interaction *PV* × *CD* on *CI*	−0.187	0.044	[−0.274, −0.101]
Interaction *CI* × *CD* on *CE*	−0.142	0.048	[−0.237, −0.048]
Index of moderated mediation	−0.119	0.034	[−0.187, −0.054]
Conditional VAF range	0.483–0.621	-	-

The contrast is informative. For Chinese and Japanese respondents, *V*_*c*_ and *V*_*a*_ dominate the conversion of perception into identification, with coefficients near 0.40 and 0.31 respectively. For German and American respondents, those coefficients shrink toward 0.27–0.29, while *V*_*f*_ and *V*_*e*_ pick up relative weight. The chi-square difference test confirms that the *V*_*c*_ and *V*_*a*_ paths differ across groups at *p* < 0.05, whereas the *V*_*e*_ and *V*_*s*_ paths are statistically invariant. Reading this against the cultural-distance moderator, we interpret the shift as evidence of route substitution: when the cultural code is less legible, audiences fall back on emotional and functional cues, exactly as the dual-route logic outlined in Section 2.3 would predict (Kim and Kim, 2022).

Figure 7 places the four country profiles in a single frame, making the route-shift pattern legible at a glance. The pooled-vs-group coefficient gaps can be summarized as (Equation 20)


$$\begin{array}{c} \Delta \gamma_{k}^{\left(j\right)}=\gamma_{k}^{\left(j\right)}-\gamma_{k}^{\left(pool\right)} \end{array}$$ (20)

(researcher-defined deviation index). Computed values place |Δγ_*c*_| at 0.047 (China, +) and 0.079 (USA, –), with |Δγ_*a*_| at 0.028 (Japan, +) and 0.055 (USA, –). Hypothesis H1, anticipating cultural and aesthetic dominance for domestic audiences, is supported. The next subsection traces how these identity outcomes feed into communication effects (Ma and Chen, 2023; Tan and Lee, 2023; Huang and Zhang, 2022).

**Figure 7:**
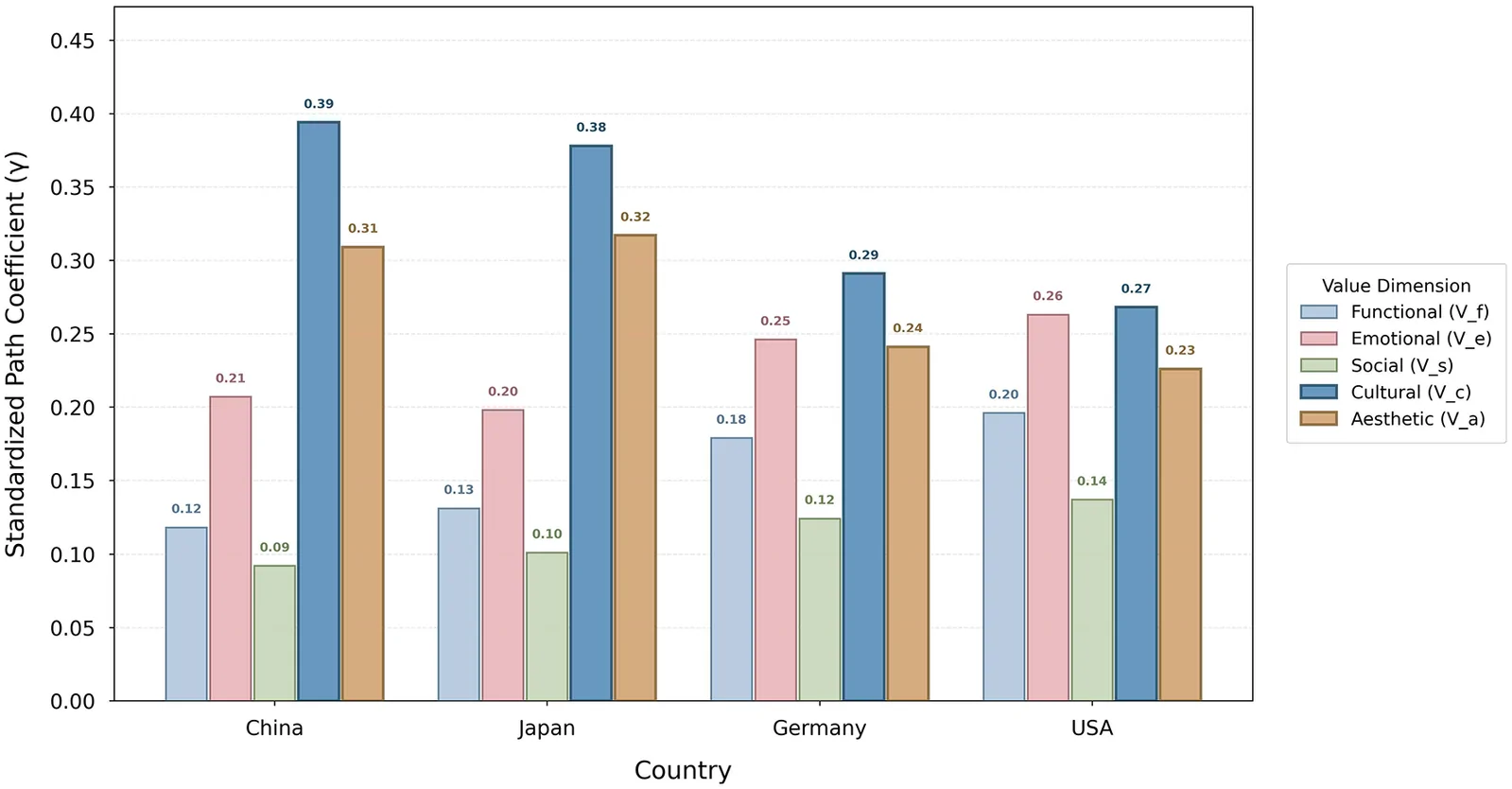
Side-by-side visualization of the five value-to-identity standardized path coefficients across the four national samples, highlighting the attenuation of cultural-value and aesthetic-value paths and the relative elevation of functional and emotional paths in the German and US groups.

### 4.3 Mediation by cultural identity and moderation by cultural distance

Having established the direct linkage from perceived value to cultural identity, the analysis turns to whether identity in turn relays value into communication outcomes, and whether cultural distance bends those pathways. A composite perceived-value index was first formed as a weighted aggregate of the five dimensions, with weights estimated from the pooled measurement model. The mediation analysis followed PROCESS Model 4 with 5,000 bias-corrected bootstrap resamples, drawing inference from 95% confidence intervals that exclude zero (Hayes, 2022). Unlike the fully standardized coefficients of Section 4.2, the mediation and moderated-mediation estimates reported in this subsection are unstandardized regression weights, because the bias-corrected bootstrap and the index of moderated mediation are defined on that metric; Table 8 is labeled accordingly, while the ratio-based VAF in Equation (23) is metric-free and thus comparable across specifications. The total, direct, and indirect effects decompose as (Equations 21, 22)


$$\begin{array}{c} c=c^{\prime }+ab \end{array}$$ (21)

$$\begin{array}{c} Indirect_{PV\to CI\to CE}=ab,\;95\%\;CI=\left[LB,UB\right] \end{array}$$ (22)

(standard Baron–Kenny / MacKinnon mediation product). For the pooled sample, the total effect of *PV* on *CE* stood at *c* = 0.612 (*p* < 0.001), with the indirect effect through *CI* equal to *ab* = 0.341 (95*%CI* : 0.2910.394) and the direct effect *c*′ = 0.271 (*p* < 0.001). The mediation share is therefore


$$\begin{array}{c} VAF=\frac{ab}{c}=\frac{0.341}{0.612}=0.557 \end{array}$$ (23)

(variance accounted for, standard Hair et al. formulation), placing the model in the partial-mediation regime rather than full mediation.

Figure 8 lays out the country-specific decomposition: the indirect share is largest for Chinese respondents (VAF = 0.621) and smallest for US respondents (VAF = 0.483), suggesting that identification carries proportionally more of the propagation impulse where cultural proximity is high.

**Figure 8:**
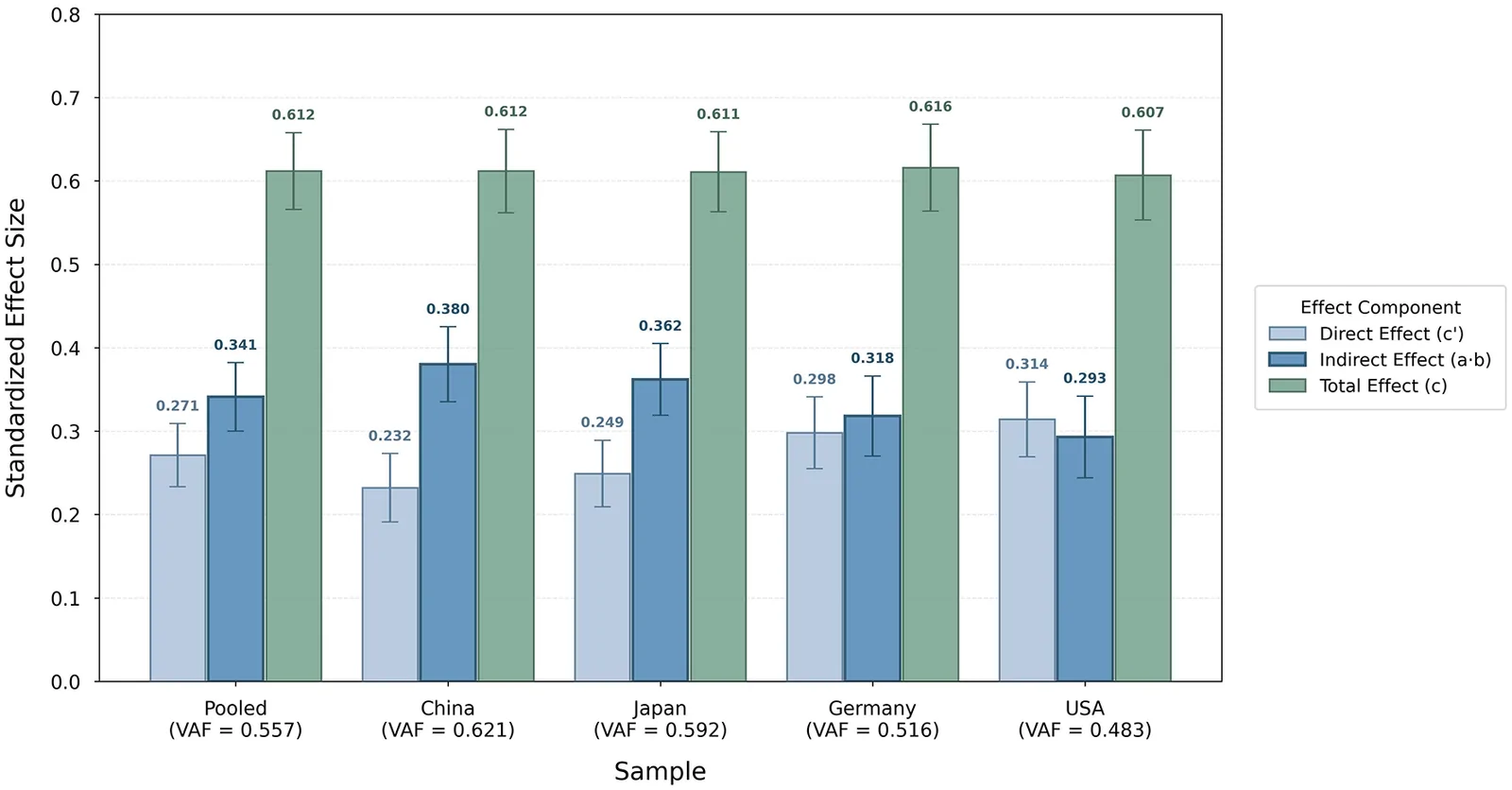
Bootstrap-based comparison of indirect, direct, and total effects of perceived value on communication effect through cultural identity, with 95% bias-corrected confidence intervals across the four national samples.

To test the moderating role of cultural distance, *CD* scores were computed as the Euclidean distance between audience country and stimulus-source country across Hofstede's six dimensions, then mean-centered before forming the latent interaction term. The moderated-mediation model takes the form (Equations 24, 25)


$$\begin{array}{c} CI=a_{0}+a_{1}PV+a_{2}CD+a_{3}\left(PV\times CD\right)+\varepsilon_{1} \end{array}$$ (24)

$$\begin{array}{c} CE=b_{0}+b_{1}CI+b_{2}PV+b_{3}\left(CI\times CD\right)+\varepsilon_{2} \end{array}$$ (25)

(standard Hayes Model 59 specification). Estimated coefficients gave *a*_3_ = −0.187 (*p* < 0.001) and *b*_3_ = −0.142 (*p* < 0.01), both indicating attenuation of the focal paths as cultural distance widens. The conditional indirect effect at ±1*SD* of *CD* is (Equation 26)


$$\begin{array}{c} \omega_{CD}=\left(a_{1}+a_{3}CD\right)\left(b_{1}+b_{3}CD\right) \end{array}$$ (26)

producing ω_−1*SD*_ = 0.412 and ω_+1*SD*_ = 0.218, with the index of moderated mediation (Equation 27)


$$\begin{array}{c} IMM=a_{3}b_{1}+a_{1}b_{3}=-0.119 \end{array}$$ (27)

reaching significance at *p* < 0.01 (standard Hayes index Hayes and Rockwood, 2020).

Simple-slope decomposition, again on the unstandardized metric, clarifies the substantive shape. At low cultural distance (−1 SD), the *PV* → *CE* slope through *CI* is steep (β = 0.487); at high cultural distance (+1 SD), it flattens markedly (β = 0.241).

As Figure 9 depicts, the two slopes converge at low perceived value but fan out sharply as perceived value rises — meaning that when audiences do not value the artifact, cultural distance scarcely matters, but when they do, the cultural gap reshapes how much of that valuation actually translates into sharing and advocacy.

**Figure 9:**
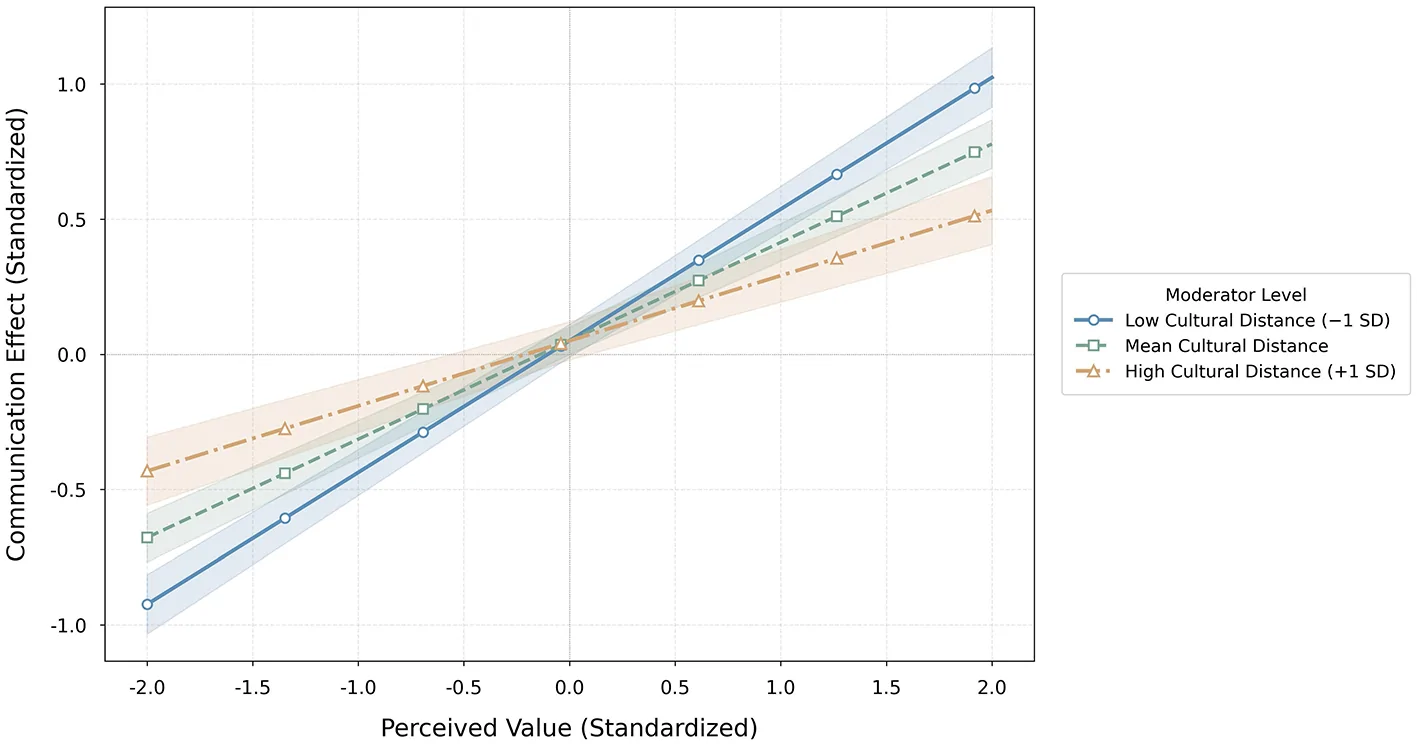
Simple-slope interaction depicting communication effect as a function of perceived value at low (−1 SD) and high (+1 SD) levels of cultural distance, showing convergence at low PV and divergence at high PV.

Table 8 consolidates the moderated-mediation evidence.

Hypotheses H3 and H4 are supported: cultural identity carries roughly half the value-to-effect transmission, and cultural distance trims that transmission as it widens (Igartua and Hayes, 2021).

## 5 Discussion

The empirical pattern that emerged from the four-country comparison invites a more interpretive reading than the path coefficients alone allow. Across all samples, audiences converge on a five-dimensional perception of digital traditional music — the structure itself travels — yet the relative weight assigned to each dimension does not. This dual finding, common structure with divergent weighting, is itself a contribution: it reframes the long-standing debate over universalism vs. relativism in cultural aesthetics, suggesting that the disagreement may concern emphasis rather than ontology. The five dimensions are evidently legible to listeners across the Confucian, Japanese, Germanic, and Anglo clusters; what shifts is the gravitational pull each dimension exerts on identification and propagation.

The differential weighting of aesthetic and cultural value across groups deserves closer scrutiny. Among Chinese and Japanese audiences, both dimensions function as deeply embedded interpretive anchors — the timbre of a guqin or the vocal grain of a Noh chant carries layered associations with ritual, lineage, and aesthetic schooling that are not reducible to surface beauty. For German and American audiences, by contrast, the same sonic events arrive without that interpretive scaffolding; what remains is largely the immediate sensory experience, which still registers (hence the persistent significance of *V*_*a*_) but lacks the cultural depth that converts perception into identification. we read this asymmetry not as evidence that foreign audiences are aesthetically deficient — they obviously are not — but as a reminder that aesthetic perception is partly historical, accumulated through repeated exposure within a tradition. Research on musical enculturation makes the mechanism explicit: listeners acquire culture-specific representations of pitch, meter, and phrase through ordinary exposure, and those representations subsequently govern what strikes them as well formed or expressive (Hannon and Trainor, 2007). Aesthetic response to an unfamiliar tradition is therefore not absent but under-scaffolded — a different diagnosis, with different practical consequences, from aesthetic indifference.

The mediating role of cultural identity speaks directly to the dual-route logic of the elaboration likelihood model. The pooled VAF of 0.557, paired with its country-level drift from 0.62 to 0.48, suggests that high cultural proximity nudges audiences toward central-route processing, where identification serves as the cognitive bridge between value and behavior; low proximity, by contrast, leaves more of the effect in the direct path, consistent with peripheral cues doing the work that identification cannot. Social identity theory anticipates as much: identification can mediate only where a salient and cognitively accessible category exists.

The moderating role of cultural distance carries practical weight. The negative index of moderated mediation tells us that simply pushing harder on perceived value will yield diminishing identification gains as cultural gaps widen. Strategies for international heritage dissemination therefore cannot rely on a single content recipe. Three implications follow, and each becomes more useful when stated concretely at the platform level and at the policy level rather than left as a principle.

The first concerns framing. Content design should be culturally adaptive rather than uniform, with minimal contextual scaffolding for high-proximity audiences and richer interpretive framing for distant ones. On a platform, this can be implemented without new production: a single guqin recording is published with two metadata-and-subtitle tracks, and the annotated version — carrying a thirty-second performer introduction and a one-line note on ritual context — is served to viewers outside the source-culture locale, reusing the switching mechanism that already exists for language subtitles. At the policy level, the same principle argues for making layered interpretive material a condition of funding: a national digitization programme can require that every publicly funded ICH recording be deposited together with a short contextual commentary in at least one additional language, in the way that community-consent documentation is already required under UNESCO-linked inventory procedures.

The second concerns balance across dimensions. Over-investing in cultural value alone leaves distant audiences with little to hold on to, whereas coupling cultural depth with emotional resonance and high production fidelity addresses the route-substitution pattern observed here. For a platform, the operational form is a production floor rather than a content quota — spatial-audio capture and high-bitrate delivery specified for every commissioned heritage title — since functional and emotional value are what carry distant audiences, and these are precisely the dimensions that our presentation manipulation moved most. For a funding body, it argues against review rubrics that score archival completeness alone; a heritage-digitization call can weight production quality and affective accessibility alongside scholarly depth, so that funded recordings do not become archives that nobody reaches.

The third concerns segmentation. Audience segmentation by cultural background, not merely by demographic band or platform behavior, should inform both recommendation pipelines and curatorial choice. Concretely, a recommender can carry a cultural-proximity feature derived from locale and listening history and use it to decide which version of an item to surface, instead of treating heritage material as one undifferentiated genre. In policy terms, cultural-diplomacy programs that currently report aggregate reach could report reach disaggregated by cultural-distance band, which would reveal whether an international touring or streaming initiative is in fact traveling beyond diaspora audiences or merely circulating within them.

At a more reflective level, the study extends perceived value theory beyond the consumer-goods context where it was originally formulated, demonstrating that the construct retains structural integrity when transplanted to heritage music while requiring an aesthetic dimension that was not present in PERVAL. Methodologically, the combination of cross-cultural psychometrics, latent moderation, and bootstrap-based moderated mediation offers a transferable template for comparative digital-humanities work. Whether the patterns hold for non-musical ICH forms — textile, foodway, ritual performance — remains an open question, and one we think worth pursuing.

## 6 Conclusion

This study set out to clarify how perceived value, cultural identity, and communication effect interconnect when intangible cultural heritage music circulates through digital channels, and how that interconnection bends across cultural boundaries. Drawing on a four-country, four-stimulus design with 1,547 valid respondents, we developed and validated a five-dimensional perceived-value scale, specified an integrative psychological mechanism model, and tested it through covariance-based SEM with multi-group invariance, bootstrap-based mediation, and latent-interaction moderation.

Three findings stand out. First, the five-dimensional structure — functional, emotional, social, cultural, and aesthetic value — holds across Confucian, Japanese, Germanic, and Anglo audiences, supporting its viability as a generalizable instrument for digital heritage research while accommodating culture-specific weightings. Second, cultural identity operates as a partial mediator carrying roughly 56% of the value-to-effect transmission in the pooled sample, with the mediated share rising for culturally proximate audiences and contracting for distant ones. Third, cultural distance moderates both legs of the mediation pathway, producing a route-substitution pattern in which cultural and aesthetic dimensions dominate identification for native audiences whereas functional and emotional dimensions gain relative weight under conditions of cultural unfamiliarity.

Theoretically, the work extends perceived value theory from its consumer-goods origins into the domain of heritage music, adds an aesthetic dimension absent from canonical instruments, and articulates a testable cross-cultural psychological mechanism linking digital presentation to propagation behavior. The model offers a transferable analytical scaffold for digital humanities work that has often lacked rigorous mediation-moderation machinery. In practical terms, the results argue for culturally adaptive content design, for reinforcement across value dimensions rather than single-dimension optimization, and for audience segmentation grounded in cultural distance rather than demographic proxies — guidance that bears on national digitization programmes and on heritage diplomacy alike.

Several limitations deserve acknowledgment. The four-country sampling, though spanning major cultural clusters, leaves substantial regional gaps — Latin American, African, and South Asian audiences are absent — so the route-substitution pattern is demonstrated across four clusters rather than established globally. The four-stimulus set, while genuinely indigenous to its source cultures, cannot exhaust the heterogeneity of traditional-music genres, and the 3 min clip length privileges short-form attention over the longer arcs typical of ritual performance.

Three constraints bear more directly on how the estimates should be read. All focal constructs are self-reported and were collected in a single sitting, which leaves the findings exposed to social-desirability and consistency pressures; the Harman test and the marker variable reported in Section 4.1 argue against a dominant method factor, but neither procedure can exclude common-method inflation of the observed associations, and both are diagnostic rather than corrective (Podsakoff et al., 2003). Recruitment through commercial online panels compounds the concern: panel members self-select, skew younger and more educated than the populations they stand in for, and non-probability samples of this kind are known to yield less accurate population estimates than probability samples (Cornesse et al., 2015). The absolute means reported here should accordingly not be treated as national norms, even though the within-study comparisons that carry the argument remain informative.

Most consequentially, the design is cross-sectional, and the mediation and moderated-mediation models are statistical rather than causal. Presentation format was randomized, so the between-condition contrasts in Section 4.1 support a causal reading; perceived value, cultural identity, and communication effect, by contrast, were measured concurrently and in a fixed order, which means the pathways traced in Sections 4.2 and 4.3 are directional hypotheses supported by the data rather than demonstrated causal sequences. Reverse and reciprocal specifications — identification heightening perceived value, prior advocacy consolidating identification — cannot be ruled out with these data, and the verbs used throughout to describe the pathways should be read in that light.

Future work might pursue four directions. Longitudinal designs would allow tracing how cultural identity consolidates or shifts over repeated digital encounters. Multimodal extensions — incorporating virtual reality, spatial audio, and haptic feedback — would test whether the route-substitution pattern survives richer sensory bandwidth. The encroachment of AI-generated content into heritage circulation raises questions about authenticity perception that the present framework can be adapted to examine. Finally, extension to non-musical ICH forms, from foodways to ritual movement, would test the boundaries of the five-dimensional value structure beyond the sonic domain.

## Data Availability

The original contributions presented in the study are included in the article/supplementary material, further inquiries can be directed to the corresponding author.
